# Mechanisms underlying the blood pressure lowering effects of dapagliflozin, exenatide, and their combination in people with type 2 diabetes: a secondary analysis of a randomized trial

**DOI:** 10.1186/s12933-022-01492-x

**Published:** 2022-04-28

**Authors:** Charlotte C. van Ruiten, Mark M. Smits, Megan D. Kok, Erik H. Serné, Daniël H. van Raalte, Mark H. H. Kramer, Max Nieuwdorp, Richard G. IJzerman

**Affiliations:** 1 Department of Internal Medicine, Diabetes Center, Amsterdam University Medical Center, Location VU University Medical Center, Amsterdam, The Netherlands; 2 Department of Vascular Medicine, Amsterdam University Medical Center, Location VU University Medical Center, Amsterdam, The Netherlands; 3 Department of Vascular Medicine, Amsterdam University Medical Center, Location AMC, Amsterdam, The Netherlands; 4 Department of Internal Medicine, Amsterdam Diabetes Center, Amsterdam University Medical Centers (Amsterdam UMC), Location VU University Medical Center (VUMC), De Boelelaan 1117 (room ZH 4A63), 1081 HV Amsterdam, The Netherlands

**Keywords:** SGLT2 inhibitor, Dapagliflozin, GLP-1 receptor agonist, Exenatide, Type 2 diabetes, Blood pressure, Autonomic balance, Hemodynamics

## Abstract

**Background:**

Sodium-glucose cotransporter-2 inhibitors (SGLT2i) and glucagon-like peptide-1 receptor agonists (GLP-1RA) lower blood pressure (BP). When SGLT2i and GLP-1RA are combined, synergistic effects on BP have been observed. The mechanisms underlying these BP reductions are incompletely understood. The aim of this study was to assess the mechanisms underlying the BP reduction with the SGLT2i dapagliflozin, GLP-1RA exenatide, and dapagliflozin-exenatide compared with placebo in people with obesity and type 2 diabetes.

**Methods:**

Sixty-six people with type 2 diabetes were randomized to 16 weeks of dapagliflozin 10 mg/day, exenatide 10 µg twice daily, dapagliflozin-exenatide, or placebo treatment. The effect of treatments on estimates of: (1) plasma volume (calculated by Strauss formula, bioimpedance spectroscopy, hematocrit, (2) autonomic nervous system activity (heart rate variability), (3) arterial stiffness (pulse wave applanometry), (4) systemic hemodynamic parameters including peripheral vascular resistance, cardiac output and stroke volume (all derived from non-invasively systemic hemodynamic monitoring), and (5) natriuresis (24-hour urine collection) were assessed after 10 days and 16 weeks of treatment.

**Results:**

After 10 days, dapagliflozin reduced systolic BP (SBP) by − 4.7 mmHg, and reduced plasma volume. After 16 weeks, dapagliflozin reduced SBP by − 4.4 mmHg, and reduced sympathetic nervous system (SNS) activity. Exenatide had no effect on SBP, but reduced parasympathetic nervous system activity after 10 days and 16 weeks. After 10 days, dapagliflozin-exenatide reduced SBP by − 4.2 mmHg, and reduced plasma volume. After 16 weeks, dapagliflozin-exenatide reduced SBP by − 6.8 mmHg, and the reduction in plasma volume was still observed, but SNS activity was unaffected.

**Conclusions:**

The dapagliflozin-induced plasma volume contraction may contribute to the initial SBP reduction, while a reduction in SNS activity may contribute to the persistent SBP reduction. Dapagliflozin-exenatide resulted in the largest decrease in SBP. The effect on plasma volume was comparable to dapagliflozin monotherapy, and SNS activity was not reduced, therefore other mechanisms are likely to contribute to the blood pressure lowering effect of this combination, which need further investigation.

*Trial registration* Clinicaltrials.gov, NCT03361098.

## Introduction

Type 2 diabetes (T2D) is associated with cardiovascular (CV) morbidity and mortality, due to a combination of effects of hyperglycemia and frequently associated obesity, dyslipidemia and hypertension. In the last decades, two novel drug classes have been developed, which reduce multiple cardiovascular risk factors: inhibitors of the sodium-glucose cotransporter 2 (SGLT2i) and glucagon-like peptide-1 receptor agonists (GLP-1RAs) [1].

SGLT2i improve CV outcomes (3-MACE), in particular (hospitalization for) heart failure, and mortality in patients with atherosclerotic disease, irrespective of the presence of diabetes [2–4], suggesting that the CV benefit occurs beyond glucose lowering. Blood pressure (BP) reduction could partially contribute to the improved CV outcomes. The systolic BP lowering effect of SLGT2i is around 4 mmHg [5]. The underlying mechanisms for the BP lowering effect remain incompletely understood, but a sustained reduced plasma volume could contribute [6, 7]. Because reductions in plasma volume and (arterial) BP occur without a rise in heart rate (HR), it has been suggested that sympathetic nervous system (SNS) activity is also reduced [3, 8–10]. Improvements in arterial stiffness and improved endothelial function have also been proposed to contribute to the long-term BP reduction [11–13].

Similar to SGLT2i, GLP-1RAs reduce cardiovascular disease (CVD) in T2D patients [14–18], and their mode of action is also not completely understood, but is likely multifactorial. GLP-1RAs reduce BP (the size of the reduction is dependent of the agent used), which may contribute to the reduction in CVD [19–21]. Mechanisms of the BP effect of GLP-1-RA may include contributions from reduced vascular resistance, natriuresis and body weight reduction [22].

Treatments that address more than one regulatory pathway are intended to enhance efficacy by synergistic responses that result from potentially engaging complementary mechanisms. Therefore, given their unique mechanisms of lowering glucose and body weight, the combination of SGLT2i and GLP-1RA appears promising [23–25]. Importantly, in DURATION-8, dapagliflozin plus exenatide resulted in a synergistic (i.e., larger than the sum of the BP reductions with both individual treatments) SBP reduction (− 4.3 mmHg) versus dapagliflozin or exenatide monotherapy after 28 weeks (− 1.8 mmHg and − 1.2 mmHg respectively) [25]. In two other trials, the addition of GLP-1RA to ongoing SGLT2i treatment also further reduced SBP (semaglutide − 4.7 mmHg, dulaglutide − 4.5 mmHg) [24, 26]. Both agents may reduce BP via similar pathways, but also potentially intervene in different regulatory pathways, which could induce a synergistic SBP reduction. Therefore, the aim of this study was to assess the mechanisms underlying the blood pressure reduction (i.e., changes in plasma volume, autonomic nervous system (ANS) activity, arterial stiffness, vascular resistance, and natriuresis) with dapagliflozin, exenatide, and dapagliflozin plus exenatide compared with placebo in people with obesity and type 2 diabetes.

## Methods

### Trial design

This was a prespecified secondary analysis of the DECREASE study: a monocenter, randomized, double-blind, placebo-controlled trial primarily designed to assess the separate and combined effects of dapagliflozin and exenatide versus placebo on activity in central reward and satiety circuits in response to food related stimuli in obese T2D subjects [27]. The study was conducted between September 2017 and May 2020 at the Amsterdam University Medical Centers, location VUmc, Amsterdam, The Netherlands. The study protocol, protocol amendments, and any other protocol-specific documents were reviewed and approved by local authorities and the ethics review board of the Amsterdam University Medical Center, location VUmc. The study complied with the Declaration of Helsinki and Good Clinical Practice guidelines and was registered at the ClinicalTrials.gov (NCT03361098).

### Participants

Participants were recruited from our outpatient clinic database and by advertisement in local newspapers. Eligible participants were men or postmenopausal women, aged 18–75 years, with a stable body weight (< 5% reported change during the previous 3 months), a BMI > 25 kg/m^2^, and diagnosed with T2D. For the current treatment of T2D metformin with or without sulfonylurea derivatives was allowed (stable dose for ≥ 3 months). HbA1c levels for participants treated with metformin monotherapy were 7–10% (53–86 mmol/mol) and for metformin plus sulfonylurea 7.5–10% (58–86 mmol/mol). Exclusion criteria were a history of severe cardiovascular, renal or liver disease, malignancies (excluding basal cell carcinoma), uncontrolled thyroid disease, the use of any centrally acting agent or oral glucocorticoids, substance abuse, neurological or psychiatric disease including eating disorders and depression and MRI contra-indications. Written informed consent was obtained from all participants.

### Study protocol

The detailed study protocol was previously published [27]. In short, participants were randomized using block randomization (1:1:1:1) to one of the four treatment groups: (1) SGLT2i dapagliflozin 10 mg/day in combination with placebo of the GLP-1RA exenatide, (2) exenatide 10 µg twice daily in combination with placebo of dapagliflozin, (3) combination of both dapagliflozin plus exenatide, or (4) double placebo. To maintain blinding throughout the study, participants were treated in a double-dummy design. There was no difference in appearance between exenatide and placebo injections or dapagliflozin or placebo tablets. Adherence was followed up by counting the remaining capsules and injection pens at all visits.

### Endpoint measurements

The study consisted of three endpoint visits: at baseline, after 10 days of treatment (short-term effects), and after 16 weeks of treatment (long-term effects). At each visit, measurements of anthropometrics were performed and blood was drawn for fasting outcome variables. On each visit, patients arrived at 8:30 am after an overnight fast at the research unit. They were instructed to refrain from vigorous physical activity and alcohol ingestion for at least 24 h, and to withhold from caffeine for more than 12 h.

Prior to each measurement patients were acclimatized for at least 10 min. All measurements were performed in the fasting state, in a semi-supine position, and in a temperature controlled room (23.0 ± 1.0 °C). Measurements were performed at the non-dominant arm comfortably placed at heart level and appropriate cuff sizes were used where applicable.

### Heart rate variability measures

Using an electrocardiogram (ECG)-equipped Nexfin device, 5-min RR-interval recordings were obtained, during which patients were instructed to breath spontaneously (range 10–18 breaths/min) and to refrain from sleeping or speaking. ECG-measurements were visually inspected and artifacts were manually corrected using linear interpolation. ECG recordings were loaded into Kubios heart rate variability (HRV) analysis software 2.2 (University of Eastern Finland, Biosignal Analysis and Medical Imaging Group, Kuopio, Finland). After additional automated low-level artifact correction and removal of trend components, Fast Fourier spectral analyses were performed to obtain normalized low frequency (LF; 0.04–0.15 Hz) and high frequency (HF; 0.15–0.5 Hz) bands, from which the low frequency/high frequency ratio (LF/HF ratio), a validated marker for cardiac sympathovagal-balance [13], was calculated. In addition, the standard deviation of the R-R intervals (SDNN), and the root mean square of successive differences (RMSSD) were calculated as measures of heart rate variability. Parasympathetic activity was assessed by RMSSD and SDNN, whereas LF/HF ratio assessed sympathovagal-balance, with LF contributing to the sympathetic activity and HF to the parasympathetic activity [28, 29].

### Systemic hemodynamic function

SBP, DBP, mean arterial pressure (MAP), and HR were determined by an automated oscillometric device (Dinamap, GE Healthcare, Little Chalfont, UK). All measurements were performed in triplicate at 1–2 min intervals, the mean of the three measurements was used for each time point. Pulse pressure (PP) was calculated by subtracting DBP from SBP. Stroke volume (SV), cardiac output (CO) and systemic vascular resistance (SVR) were calculated from noninvasive beat-to-beat finger arterial photoplethysmography BP measurements (Nexfin, Amsterdam, The Netherlands). The finger BP-measurements were performed over a period of 30s, and a mean was derived using dedicated software (Nexfin@PC version 2.0, BM Eye, Amsterdam, The Netherlands).

### Plasma volume

Participants collected 24 h urine samples that ended on the night before testing. Urinary excretion of volume, sodium and glucose were subsequently measured. Bioimpedance spectroscopy (BIS) (ImpediMed Limited, Pinkenba, Queensland, Australia) for assessment of extra- and intracellular volume was performed. Blood was drawn from the cubital vein to measure hematocrit. The percentage change in estimated plasma volume (ePV) was calculated by the Strauss formula [30–32]:

$$\left( {\left( {\left( {{{{\text{Hb}}_{{{\text{baseline}}}} } \mathord{\left/ {\vphantom {{{\text{Hb}}_{{{\text{baseline}}}} } {{\text{Hb}}_{{{\text{end}}}} }}} \right. \kern-\nulldelimiterspace} {{\text{Hb}}_{{{\text{end}}}} }}} \right) \times \left( {{{\left( {{\text{1}}00 - {\text{ Ht}}_{{{\text{end}}}} } \right)} \mathord{\left/ {\vphantom {{\left( {{\text{1}}00 - {\text{ Ht}}_{{{\text{end}}}} } \right)} {\left( {{\text{1}}00{\text{ }} - {\text{ Ht}}_{{{\text{baseline}}}} } \right)}}} \right. \kern-\nulldelimiterspace} {\left( {{\text{1}}00{\text{ }} - {\text{ Ht}}_{{{\text{baseline}}}} } \right)}}} \right)} \right) - {\text{1}}} \right) \times {\text{100}}.$$

### Pulse wave analysis

Pulse wave analysis (PWA) was performed at the radial artery using applanation tonometry with a high fidelity micromanometer (SPT-301; Millar Instruments, Houston, Texas, USA) coupled to a SphygmoCor^®^ System (Atcor Medical, West Ryde, Australia) to assess arterial stiffness [33, 34]. The average of two recordings (of ≥ 12 s) was used, which needed to have adequate pulse wave profiles and a high-quality control, defined as an in-device quality index of more than 80%. The central aortic pressure wave form was derived from the radial artery waveform using the software’s mathematical transfer function [33, 34]. The augmentation index (an indicator of arterial stiffness) was calculated as the augmentation pressure, that is, the pressure of the second systolic peak minus the pressure at the inflection point, expressed as percentage of the pulse pressure and normalized for a HR of 75 bpm (AIX@HR75).

### Statistical analyses

All statistical analyses were performed using SPSS 26.0 (IBM SPSS). Linear mixed models (LMM) were performed to investigate treatment effects versus placebo over time in the per protocol population. For the change in BP, within group differences were also calculated. Treatment allocation was set as independent variable in dummy variables, and the endpoint of interest as dependent variable. Time was added as fixed factor. The treatment allocation interaction with time, as well as a random intercept were included in the model. Also, corresponding baseline values were added as independent variable. *P* value < 0.05 was considered as statistically significant. No corrections for multiple testing were performed, because we felt that false-negative findings would be more troublesome for this mechanistic study compared with false-positive findings.

## Results

### Baseline characteristics

**Fig. 1:**
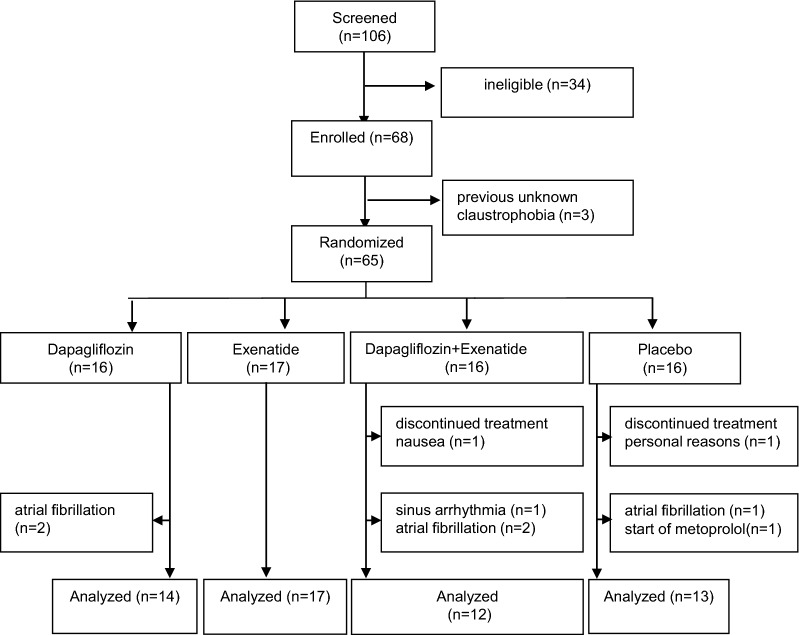
Flow chart inclusion

One hundred and six people were screened, of whom 68 were included. Before randomisation three patients were excluded due to undiagnosed claustrophobia (important for primary endpoint). Two patients dropped out just before the last test visit, one because of personal reasons (placebo group), the other one because of ongoing nausea (combination group). In total, seven patients were excluded in the analysis for ANS activity; five patients with atrial fibrillation (n = 1 dapagliflozin, n = 3 combination, n = 1 placebo), one patient with severe sinus arrhythmia in the combination group, and one patient who started metoprolol during the study in the placebo group (Fig. 1). Baseline characteristics of the per protocol population (n = 56) were well balanced between treatment (Table 1). For all participants, the medications used at baseline, remained unchanged during the study. Medication adherence for dapagliflozin (or placebo) was 99.0% and for exenatide (or placebo) 97.5%.

**Table 1:** Baseline characteristics

	Dapagliflozin (n = 14)	Exenatide (n = 17)	Dapagliflozin + exenatide (n = 12)	Placebo (n = 14)
Age (years)	63.6 (8.5)	65.0 (5.8)	62.8 (7.7)	60.9 (6.9)
Female (n (%))	4 (28.6)	6 (35.3)	4 (33.3)	4 (28.6)
Weight (kg)	98.5 (15.6)	96.6 (13.3)	90.5 (13.3)	99.1 (21.9)
BMI (kg/m^2^)	31.8 (3.4)	32.7 (5.1)	30.4 (3.8)	31.5 (5.9)
Diabetes duration (years)	7.0 [5.0,12.0]	10.0 [6, 18]	6.5 [3.0,7.0]	9.0 [7,11.0]
HbA1c (%) (mmol/mol)	7.7 (0.5) 60.7 (5.8)	7.9 (0.8) 65.0 (11.1)	8.3 (1.3) 66.9 (14.2)	8.0 (1.0) 65.2 (11.7)
Hematocrit (%)	43.6 (0.9)	44.1 (0.8)	43.3 (0.5)	43.3 (0.9)
SBP (mmHg)	134.7 (2.2)	132.1 (2.7)	127.0 (2.9)	132.6 (3.3)
DBP (mmHg)	81.0 (1.5)	81.0 (1.8)	78.4 (2.0)	81.3 (1.7)
HR (bpm)	65.3 (2.9)	71.0 (2.4)	71.4 (2.3)	68.5 (2.4)
Pulse Pressure (mmHg)	53.6 (2.5)	51.1 (2.2)	48.7 (5.4)	51.3 (2.8)
Use of (n (%))				
Metformin	14 (100)	17 (100)	12 (100)	14 (100)
SU derivative	4 (28.6)	6 (35.3)	3 (25.0)	8 (57.0)
Beta blocker	4 (28.6)	4 (23.5)	1 (8.3)	2 (14.2)
RAS inhibition	5 (35.7)	12 (70.6)	6 (54.5)	9 (64.3)
ACE inhibitor	2 (40.0)	8 (66.7)	4 (66.7)	6 (66.7)
ARB	3 (60.0)	4 (33.3)	2 (33.3)	3 (33.3)
Urine volume (ml/24 h)	1886.0 (836.4)	2216.4 (205.8)	2022.9 (848.4)	1610.3 (657.9)
Sodium excretion (mmol/24 h)	151.0 (69.0)	179.7 (59.2)	148.8 (67.5)	164.9 (68.2)

Baseline characteristics of the per protocol population. Data are means ± SD or median [interquartile range] for continuous metrics, and number (%) for categorical characteristics

BMI, body mass index; SBP, systolic blood pressure; DBP, diastolic blood pressure; HR, heart rate (beats per minute); SU, Sulfonylurea; ACE, angiotensin converting enzyme; ARB, angiotensin-II receptor blocker

### Glycemic and body weight effects

All active treatments resulted in significant reductions in HbA1c and body weight, with the largest reduction in the dapagliflozin plus exenatide group compared with placebo after adjustment for baseline (HbA1c%: − 1.2 ± 0.19%; body weight: − 2.8 ± 0.5 kg) [27].

### Blood pressure and heart rate

After 10 days, dapagliflozin treatment resulted in SBP reduction of − 4.7 ± 2.1 mmHg (p = 0.047), and after 16 weeks in a SBP reduction of − 4.4 ± 2.7 mmHg (p = 0.13). Exenatide had no significant effect on SBP. After 10 days, dapagliflozin-exenatide reduced SBP by − 4.2 ± 1.9 mmHg (p = 0.046), and after 16 weeks by − 6.8 ± 2.4 mmHg (p = 0.015). Placebo had no significant effect on SBP (Table 2). After correction for placebo values dapagliflozin numerically reduced BP after 10 days of treatment (− 4.5 ± 2.9 mmHg, p = 0.13). Exenatide had no effect on SBP. Dapagliflozin plus exenatide resulted in the largest BP reduction of − 6.4 ± 3.0 mmHg (p = 0.039) after 10 days, and − 6.7 ± 3.1 mmHg (p = 0.033) after 16 weeks. HR was numerically increased by exenatide and dapagliflozin plus exenatide, but not by dapagliflozin (Table 2).

**Table 2:** Changes in blood pressure and heart rate

	Dapagliflozin		Exenatide		Dapagliflozin + exenatide		Placebo	
	1.5 weeks	16 weeks	1.5 weeks	16 weeks	1.5 weeks	16 weeks	1.5 weeks	16 weeks
Blood pressure and heart rate								
SBP (mmHg)								
Within groups	− 4.7 ± 2.1*	− 4.4 ± 2.7	− 0.6 ± 1.7	− 1.3 ± 2.1	− 4.2 ± 1.9*	− 6.8 ± 2.4*	− 0.9 ± 2.3	− 1.4 ± 2.4
Between groups	− 4.5 ± 2.9	− 1.8 ± 3.0	0.45 ± 2.8	0.5 ± 2.8	− 6.4 ± 3.0*	− 6.7 ± 3.1*		
DBP (mmHg)	− 1.5 ± 1.8	0.5 ± 1.8	− 0.1 ± 1.7	1.8 ± 1.8	− 2.3 ± 1.9	− 1.7 ± 1.9		
Heart rate (bpm)	0.2 ± 2.8	2.4 ± 2.8	3.9 ± 2.7	3.0 ± 2.8	3.4 ± 2.9	3.2 ± 2.9		

Linear mixed models were used to compare baseline corrected treatment effects with placebo (mean ± SE). For systolic blood pressure, within group differences and between group differences are displayed

*P < 0.05 for differences in change during treatments compared with placebo

SBP, systolic blood pressure; DBP diastolic blood pressure

### Indicators of plasma volume

Dapagliflozin compared with placebo tended to increase urine volume by 419.4 ± 220.6 ml/24 h (p = 0.06; 10 days), but not after 16 weeks (244.1 ± 224.1 ml/24 h, p = 0.28). Dapagliflozin compared with placebo showed a small non-significant increase in hematocrit (1.2 ± 0.8%, p = 0.17). Dapagliflozin compared with placebo decreased extracellulair fluid (− 1.1 ± 0.4 L, p = 0.013) after 10 days, but not after 16 weeks (− 0.6 ± 0.4 L, p = 0.17). Dapagliflozin compared with placebo reduced estimated plasma volume (ePV) by − 3.5% (95% CI − 7.7 to − 0.7, p = 0.001) after 10 days, but not after 16 weeks (− 1.3%, 95% CI − 4.3 to 6.7, p = 0.55) (Table 3).

**Table 3:** Measures of cardiovascular function

	Dapagliflozin		Exenatide		Dapagliflozin + Exenatide	
	1.5 weeks	16 weeks	1.5 weeks	16 weeks	1.5 weeks	16 weeks
Hemodynamics						
Stroke volume (ml)	− 3.6 ± 4.2	− 3.0 ± 4.5	− 5.6 ± 4.0	− 10.4 ± 4.2*	− 6.0 ± 4.4	− 7.2 ± 4.4
Cardiac output (l/min)	− 0.1 ± 0.3	− 0.02 ± 0.3	− 0.04 ± 0.3	− 0.1 ± 0.3	− 0.2 ± 0.3	− 0.2 ± 0.3
Systemic vascular resistance (dyn/s/cm)	− 8.2 ± 83.1	− 86.4 ± 88.7	30.2 ± 79.2	− 25.4 ± 82.0	5.3 ± 86.1	− 44.0 ± 89.0
Arterial stiffness						
AIX@HR75	− 2.6 ± 2.7	2.3 ± 2.7	2.2 ± 2.6	2.3 ± 2.6	0.2 ± 2.8	3.9 ± 2.9
Pulse pressure (mmHg)	− 3.4 ± 2.1	0.3 ± 2.1	− 0.07 ± 2.0	− 1.0 ± 2.0	− 3.9 ± 2.2	0.5 ± 2.2
Autonomic function						
LF/HF ratio	− 0.09 ± 0.6	− 1.1 ± 0.6	0.1 ± 0.5	− 0.3 ± 0.5	0.7 ± 0.6	− 0.4 ± 0.6
SDNN (ms)	− 2.3 ± 4.0	− 3.8 ± 3.9	− 2.5 ± 3.5	− 8.1 ± 3.6*	− 9.6 ± 3.7*	− 8.7 ± 4.0*
RMSSD (ms)	− 0.9 ± 4.5	− 0.9 ± 4.7	− 5.1 ± 4.2	− 8.4 ± 4.4	− 11.4 ± 4.6*	− 10.7 ± 4.8*
Hematocrit, body water, urinary volume, glucose and sodium excretion						
Change in ePV (%)	− 3.5 ± 1.5**	− 1.3 ± 2.0	1.0 ± 1.5	2.5 ± 2.0	− 4.2 ± 1.7*	− 3.9 ± 2.1*
Hematocrit (%)	1.2 ± 0.8	− 0.01 ± 0.9	− 0.8 ± 0.8	− 2.4 ± 0.9**	2.0 ± 0.9*	1.3 ± 0.9
Extracellular fluid (L)	− 1.1 ± 0.4**	− 0.6 ± 0.4	− 0.1 ± 0.4	− 0.3 ± 0.4	− 0.7 ± 0.4	− 0.4 ± 0.4
Intracellular fluid (L)	− 0.4 ± 0.5	− 0.7 ± 0.6	0.2 ± 0.5	− 0.4 ± 0.5	− 0.3 ± 0.6	− 0.2 ± 0.6
Urine volume (l/24 h)	419.4 ± 220.6	244.1 ± 224.1	− 27.0 ± 216.3	− 429.6 ± 222.4	391.9 ± 231.5	− 61.8 ± 239.3
Glucose excretion (mmol/24 h)	500.7 ± 4.7***	455.7 ± 74.7***	− 7.0 ± 70.7	− 47.4 ± 71.9	313.0 ± 79.8***	263.3 ± 82.5**
Sodium excretion (mmol/24 h)	16.4 ± 24.9	20.4 ± 25.3	− 3.7 ± 23.7	− 27.7 ± 24.7	31.7 ± 26.0	− 12.7 ± 26.9

Linear mixed models were used to compare baseline corrected treatment effects with placebo (mean ± SE)

*P < 0.05; **P < 0.01; ***P < 0.001 for differences in change during treatments compared with placebo

ePV, estimated plasma volume was calculated with the Strauss formula

After 10 days, exenatide compared with placebo had no effect on any of the 24 h urine measurements. After 16 weeks of treatment, exenatide compared with placebo tended to decrease urine volume by − 429.6 ± 222.4 ml/24 h (p = 0.056). Hematocrit was decreased by − 2.4 ± 0.9% (p = 0.006). Extracellular fluid and ePV were unaffected (Table 3).

**Fig. 2:**
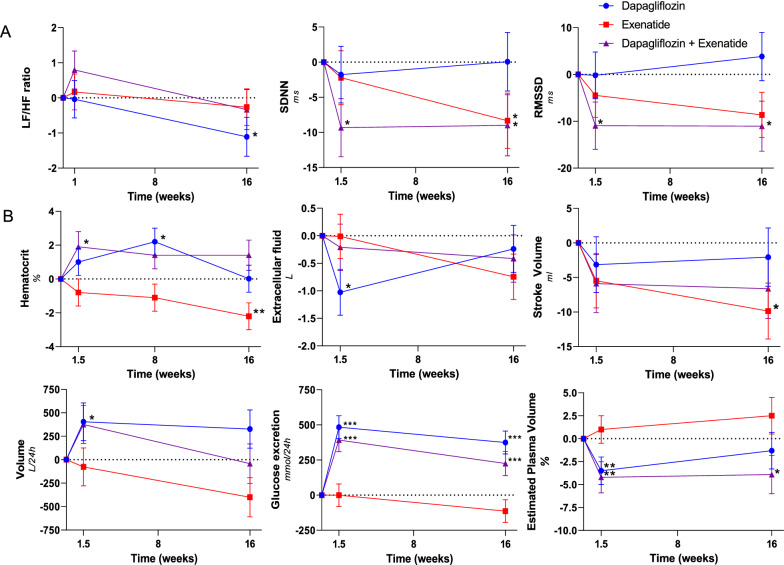
Linear mixed models were used to compare baseline corrected treatment effects with placebo. Changes in **A** autonomic balance, **B** indirect measurements of plasma volume after dapagliflozin (blue circle), exenatide (red square), dapagliflozin plus exenatide (purple upward triangle after 10 days, and 16 weeks of treatment. Data points represent mean with SEM. Statistically significant mean differences of treatments compared with placebo corrected for baseline values are indicated as *(*P* < 0.05), **(*P* < 0.01), ***(*P* < 0.001)

Dapagliflozin plus exenatide compared with placebo tended to increase urine volume after 10 days (391.9 ± 231.5 ml/24 h, p = 0.09), but not after 16 weeks of treatment. Hematocrit was increased by 2.0 ± 0.9% (p = 0.029) after 10 days, but only showed a small numerical increase after 16 weeks (1.3 ± 0.9%, p = 0.16). Dapagliflozin plus exenatide compared with placebo numerically reduced extra-cellular fluid after 10 days (− 0.7 ± 0.4, p = 0.13), but not after 16 weeks. Dapagliflozin plus exenatide compared with placebo reduced ePV by − 4.2% (95% CI − 8.8 to − 0.4, p = 0.014) after 10 days, and by − 3.9% (95% CI − 9.8 to − 1.9, p = 0.045) after 16 weeks (Table 3; Fig. 2).

### Autonomic nervous system activity

After 10 days, dapagliflozin compared with placebo had no effect on LF/HF ratio. Dapagliflozin compared with placebo tended to reduce LF/HF ratio by − 1.1 ± 0.6 (p = 0.06) after 16 weeks (an indicator of reduced sympathetic activity) (Table 3; Fig. 2). No effect on SDNN or RMSSD was observed.

After 10 days and 16 weeks, exenatide compared with placebo had no effect on LF/HF ratio. After 16 weeks, exenatide significantly reduced SDNN (− 8.1 ± 3.6, p = 0.027) and tended to reduce RMSSD (− 8.4 ± 4.4, p = 0.058) (an indicator of reduced parasympathetic activity) (Table 3; Fig. 2).

Although dapagliflozin plus exenatide compared with placebo had no effect on LH/HF ratio after 10 days and 16 weeks, dapagliflozin plus exenatide reduced RMSSD (10 days: − 11.4 ± 4.6 p = 0.015; 16 weeks: − 10.7 ± 4.8 p = 0.029) and SDNN (10 days: − 9.6 ± 3.7 p = 0.012; 16 weeks: − 8.7 ± 3.9 p = 0.030) (an indicator of reduced parasympathetic activity) (Table 3; Fig. 2).

### Systemic hemodynamic function

Dapagliflozin compared with placebo had no effect on SV, CO or SVR. Exenatide compared with placebo reduced SV by − 10.4 ± 4.2 ml (p = 0.014) after 16 weeks of treatment. CO and SVR were unaffected by exenatide treatment. Dapagliflozin plus exenatide compared with placebo tended to reduce SV by − 7.2 ± 4.4 ml (p = 0.11) after 16 weeks of treatment (Table 3; Fig. 2). CO and SVR were unaffected in the combination group.

### Arterial stiffness

None of the treatments had a statistical significant effect on arterial stiffness (AIX@HR75 or PP) (Table 3).

## Discussion

This secondary prespecified analysis of a randomized double-blind placebo controlled trial investigated potential mechanisms underlying the BP reduction with SGLT2i and GLP-1RA, and their combination, in obese people with type 2 diabetes. We observed that dapagliflozin reduced SBP, and combination of dapagliflozin and exenatide caused a superior effect on SBP lowering, which is in line with previous studies [24–26, 35, 36]. Dapagliflozin induced plasma volume contraction after 10 days of treatment, which could contribute to the acute drop in SBP. After 16 weeks of treatment, measures of plasma volume returned to baseline levels, but dapagliflozin reduced SNS activity, possibly contributing to the persistent SBP reduction. Dapagliflozin plus exenatide also induced plasma volume contraction after 10 days, contributing to the acute drop in SBP, and to a lesser extent after 16 weeks of treatment, possibly contributing to the persistent reduction in SBP. However, after 16 weeks of treatment, there was no reduction in SNS activity in the combination group, suggesting that other mechanisms contribute to the synergistic SBP reduction after 16 weeks of treatment.

We investigated underlying mechanisms which could explain the decrease in BP with dapagliflozin. SGLT2i may induce plasma volume contraction, leading to reductions in cardiac pre- and after-load [6]. Sodium- and glucose-excretion may contribute to the acute plasma volume contraction. However, observed natriuresis with SGLT2i is modest when compared with diuretics, and is transient, as is returns to pretreatment levels within days [37]. The osmotic diuretic effect induced by glucosuria may not be the only contributing factor to plasma volume contraction, as similar changes in plasma volume are observed in individuals with minimal glucosuria [38, 39]. In the current study dapagliflozin reduced ePV after 10 days of treatment. In addition, dapagliflozin significantly increased glucose excretion, and numerically increased 24 h urinary volume, increased hematocrit, and decreased extracellular fluid after short-term treatment. This plasma volume contraction however, was no longer observed after 16 weeks of treatment. This suggest that plasma volume contraction contributes in particular to the acute drop in SBP with SGLT2i treatment. Although the effect on ePV in the current study was larger, these findings are in line with a previous study, in which no effect on natriuresis (after standardized sodium intake), and no significant prolonged changes in plasma volume (directly measured with indocyanine green indicator dilution method) were observed [40].

A reduction in BP and diuretic actions usually promote baroreflex-mediated increase in SNS activity, leading to an increase in heart rate. SGLT2i however, reduce BP without an increase in heart rate, which may suggest dampening of SNS activity [9]. After 10 days and 16 weeks of treatment we indeed found no reflex-mediated sympathetic activation. In line with these results, a previous study showed that 5 days of empagliflozin did not affect muscle SNS activation despite lowering BP in T2D patients [8]. In addition, another study found that 12 weeks of dapagliflozin compared with gliclazide reduced SBP, without affecting HR or SNS activity in metformin treated T2D patients [10]. Interestingly, we even found a reduction in SNS activity after 16 weeks of treatment. These findings are in line with data from animal models [41]. It could be suggested that a reduction in SNS activity contributes to the long-term BP reduction, as we only observed a reduction after 16 weeks of treatment. Improvements in glucose, insulin resistance, and hyperinsulinemia may have contributed to the reduction in SNS activity [42, 43]. Whether the effect of dapagliflozin on SNS activity is independent of these improvements needs further investigation.

Dapagliflozin plus exenatide resulted in the largest BP reduction which is in line with large combination trials [24–26]. The combination of dapagliflozin and exenatide reduced ePV, and tended to reduce SV, accompanied by increases in hematocrit, 24 h urinary volume and glucose excretion, reflecting plasma volume contraction after short-term treatment. After 16 weeks, the combination reduced ePV, but the other parameters were unaffected. As with dapagliflozin monotherapy, plasma volume contraction may contribute mainly to the acute drop in SBP, and to a lesser extent to the persistent SBP reduction. Interestingly, the dapagliflozin-induced reduction in SNS activity after 16 weeks was not observed in combination therapy. Instead, PNS activity was reduced, as was observed with exenatide monotherapy. It could be suggested that combination with exenatide blunted the reduction in SNS activity observed with dapagliflozin, and therefore the potential contribution of a reduced SNS activity on BP reduction. However, the combination group showed the largest SBP reduction after 16 weeks of treatment, suggesting that other mechanisms than plasma volume contraction and SNS reduction could contribute to the observed synergistic reduction in SBP with the combination therapy of SGLT2i and GLP-1RA [25]. We suggest that non osmotic sodium storage, changes in baroreceptor reflex set point/renal sympathetic nerve activity, or a central neural pathway may play an important role.

A reduction in arterial stiffness could also contribute to BP lowering effect of SGLT2i. Some studies showed a reduction in arterial stiffness, measured as augmentation index or as carotid-femoral pulse-wave velocity after acute or chronic SGLT2i treatment in patients with T2D [10, 44–46]. In this study we found no reduction in arterial stiffness (measured as augmentation index) with dapagliflozin or dapagliflozin plus exenatide. It should be noticed that pulse wave velocity is a more sensitive marker to detect changes in arterial stiffness in older individuals and might have been a better measure in our population [47].

Exenatide numerically reduced SBP from baseline (− 1.3 mmHg). Although some GLP-1RA induce more SBP reduction [48], the SBP reduction in this study is slightly less than the modestly observed SBP reduction with exenatide in larger trials [20, 49, 50]. In this study baseline SBP was relatively low [51]. In our study exenatide had no effect on natriuresis, SNS activity, or arterial stiffness, which is in line with previous studies [52–54]. SV decreased without a change in CO, which could suggest systemic vasodilation with subsequent reflex tachycardia to maintain stable perfusion. However, in this study non-invasively measured vascular resistance was not affected, but had a large standard deviation, which suggests the need for further research in a larger sample. The observed increase in HR could reduce ventricular filling time which would reduce SV, while keeping cardiac output unaffected [55]. This is in line with a previous study, in which 12 weeks of liraglutide also increased heart rate, while reducing systolic blood pressure and stroke volume, whereas vascular resistance and cardiac output where unaffected [54]. This suggest that other mechanisms, such as changes in neuroendocrine hormones (i.e. angiotensin II, ANP), or a central neural pathway may contribute to BP lowering effect of GLP-1RA [56, 57]. The increase in HR did not reach statistical significance, but the point estimate was comparable to the average increase of 2–3 bpm found in other trials [20]. Although SNS activity was unchanged, we did find a reduction in PNS activity, which may contribute to the increase in HR, confirming animal models in which a significant depression of parasympathetic modulation of heart rate was found [58, 59]. A direct effect of GLP-1RA on sino-atrial node cells was also a hypothesized mechanisms for the increase in HR, and recently demonstrated in a mice model [59]. However, cardiac GLP-1 receptor circuits controlling HR required neural inputs, as the increase in HR was not observed in isolated perfused heart or mouse atria ex vivo [59]. Therefore it could be suggested that both, direct effects on the GLP-1 receptor and reduced PNS activity contribute to the increase in HR.

While the double-blinded, double-dummy, randomized 4-armed design is a major strength, this study also has some limitations. First, although this was a mechanistic prespecified secondary outcome, the sample size was small. Due to limited statistical power, this may have led to under detection of potential changes. Therefore, the results should be interpret with caution, and be considered hypothesis generating only. A dedicated clinical trial with more power is required to confirm these findings. Second, some patients used beta blockers and RAAS inhibitors. Although these medications were equally distributed among the groups, and discontinued at the day of the measurements, they may have influenced the effect on blood pressure reduction. However, the high percentage of patients using blood pressure lowering medication is in line with clinical practice and previous clinical trials in patients with diabetes. Third, although 24 h urine collection is the gold standard method to measure sodium excretion, measurements during standardized sodium intake and multiple 24 h collections may have been more precise [60]. However, the study might have been too short of duration to detect changes in arterial stiffness. Systemic hemodynamic parameters were calculated from non-invasive pulse pressure measurement devices, but these are well validated against intra-arterial measurements [61].

## Conclusions

In conclusion, our findings suggest that the dapagliflozin-induced plasma volume contraction contributes to the initial measured blood pressure reduction, while a reduction in SNS activity may contribute to the persistent blood pressure reduction. Combination therapy with dapagliflozin plus exenatide resulted in the largest decrease in blood pressure. However, as the effect on plasma volume was comparable to dapagliflozin monotherapy, and SNS activity was not reduced, other mechanisms are likely to contribute to the blood pressure lowering effect of this combination, and need further investigation.

## Data Availability

The datasets used and/or analyzed during the current study are available from the corresponding author on reasonable request.

## Abbreviations

SGLT2i: Sodium-glucose cotransporter-2 inhibitors
GLP-1: RA glucagon-like peptide-1 receptor agonist
CV: Cardiovascular
T2D: Type 2 diabetes
ANS: Autonomic nervous system
PNS: Parasympathetic nervous system
SNS: Sympathetic nervous system
BMI: Body mass index
SU: Sulfonylurea derivatives
ACE: Angiotensin converting enzyme
ARB: Angiotensin-II receptor blocker
RAAS: Renin–angiotensin–aldosterone system
ECG: Electrocardiogram
HRV: Heart rate variability
LF/HF ratio: Low frequency/high frequency ratio
SDNN: Standard deviation of the R-R intervals
RMSSD: Root mean square of successive differences
BP: Blood pressure
SBP: Systolic blood pressure
DBP: Diastolic blood pressure
MAP: Mean arterial pressure
HR: Heart rate
PP: Pulse pressure
SV: Stroke volume
CO: Cardiac output
SVR: Systemic vascular resistance
BIS: Bioimpedance spectroscopy
ePV: Estimated plasma volume
PWA: Pulse wave analysis
AIX@HR75: Augmentation index normalized for a heart rate of 75 beats per minute
LMM: Linear mixed models
